# Neurobehavioral function and low-level exposure to brominated flame retardants in adolescents: a cross-sectional study

**DOI:** 10.1186/1476-069X-11-86

**Published:** 2012-11-14

**Authors:** Michał Kiciński, Mineke K Viaene, Elly Den Hond, Greet Schoeters, Adrian Covaci, Alin C Dirtu, Vera Nelen, Liesbeth Bruckers, Kim Croes, Isabelle Sioen, Willy Baeyens, Nicolas Van Larebeke, Tim S Nawrot

**Affiliations:** 1 Centre for Environmental Sciences, Hasselt University, Diepenbeek, Belgium; 2 Department of Neurology, Sint Dimphna Hospital, Geel, Belgium; 3 Flemish Institute for Technological Research, Environmental Risk and Health, Mol, Belgium; 4 Department of Biomedical sciences, University of Antwerp, Antwerp, Belgium; 5 Toxicological Center, University of Antwerp, Antwerp, Belgium; 6 Department of Health, Provincial Institute for Hygiene, Antwerp, Belgium; 7 Interuniversity Institute for Biostatistics and Statistical Bioinformatics, Hasselt University, Diepenbeek, Belgium; 8 Department of Analytical and Environmental Chemistry, Brussels Free University (VUB), Brussels, Belgium; 9 Department of Public Health, Ghent University, Ghent, Belgium; 10 Department of Radiotherapy and Nuclear Medicine, University Ghent, Ghent, Belgium; 11 School of Public Health, Occupational and Environmental Medicine, KULeuven, Leuven, Belgium

**Keywords:** Brominated flame retardants, PBDE, TBBPA, HBCD, Neurotoxicity, Neurobehavioral function, Cognitive function, Cognition, Adolescents

## Abstract

**Background:**

Animal and in vitro studies demonstrated a neurotoxic potential of brominated flame retardants, a group of chemicals used in many household and commercial products to prevent fire. Although the first reports of detrimental neurobehavioral effects in rodents appeared more than ten years ago, human data are sparse.

**Methods:**

As a part of a biomonitoring program for environmental health surveillance in Flanders, Belgium, we assessed the neurobehavioral function with the Neurobehavioral Evaluation System (NES-3), and collected blood samples in a group of high school students. Cross-sectional data on 515 adolescents (13.6-17 years of age) was available for the analysis. Multiple regression models accounting for potential confounders were used to investigate the associations between biomarkers of internal exposure to brominated flame retardants [serum levels of polybrominated diphenyl ether (PBDE) congeners 47, 99, 100, 153, 209, hexabromocyclododecane (HBCD), and tetrabromobisphenol A (TBBPA)] and cognitive performance. In addition, we investigated the association between brominated flame retardants and serum levels of FT3, FT4, and TSH.

**Results:**

A two-fold increase of the sum of serum PBDE’s was associated with a decrease of the number of taps with the preferred-hand in the Finger Tapping test by 5.31 (95% CI: 0.56 to 10.05, p = 0.029). The effects of the individual PBDE congeners on the motor speed were consistent. Serum levels above the level of quantification were associated with an average decrease of FT3 level by 0.18 pg/mL (95% CI: 0.03 to 0.34, p = 0.020) for PBDE-99 and by 0.15 pg/mL (95% CI: 0.004 to 0.29, p = 0.045) for PBDE-100, compared with concentrations below the level of quantification. PBDE-47 level above the level of quantification was associated with an average increase of TSH levels by 10.1% (95% CI: 0.8% to 20.2%, p = 0.033), compared with concentrations below the level of quantification. We did not observe effects of PBDE’s on neurobehavioral domains other than the motor function. HBCD and TBBPA did not show consistent associations with performance in the neurobehavioral tests.

**Conclusions:**

This study is one of few studies and so far the largest one investigating the neurobehavioral effects of brominated flame retardants in humans. Consistently with experimental animal data, PBDE exposure was associated with changes in the motor function and the serum levels of the thyroid hormones.

## Background

Brominated flame retardants (BFR) are chemicals widely used in a variety of household and commercial products including plastics, electric equipment, textiles, and polyesters in order to prevent fire [1,2]. Many of them bioaccumulate in the environment and have been found in water, air, biota, human tissues, breast milk, and blood [3-6]. House dust and food represent two important sources of human exposure [5,7].

A number of animal studies showed effects of a prenatal and postnatal exposure to BFR on neurodevelopment and were recently reviewed. [8-10] Neurobehavioral effects during juvenile development or adulthood have been observed in rodents after a brief postnatal exposure to polybrominated diphenyl ethers (PBDE) 47 [11,12], 99 [11,13-16], 153 [17], 203 [18], 206 [18], 209 [19-22], the commercial PBDE mixture DE-71 [23,24], and hexabromocyclododecane (HBCD) [25,26], a chronic perinatal exposure to PBDE-47 [27,28] and PBDE-99 [29], and an acute prenatal exposure to PBDE-99 [30]. Detrimental effects of PBDE exposure on neurodevelopment have also been reported in zebrafish [31,32]. Changes in the motor activity have been most frequently studied and best documented [8,10]. Also in vitro studies support the hypothesis of neurotoxicity of BFR. PBDE congeners were capable of inducing oxidative stress [33-35] and apoptosis [33,34] in cultured neurons.

Despite the fact that the first results of the experimental animal studies suggesting a neurotoxic potential of BFR were available more than 10 years ago, the effects in humans have not been extensively investigated to date. Three small prospective studies [36-38] evaluated the effects of a perinatal exposure to BFR on neurobehavioral function in children. Concentrations of several PBDE congeners in umbilical cord blood of newborns showed an association with indicators of neurodevelopment in early childhood. [37] In another study [36], consistent neurodevelopmental effects at the age of 8–12 months of the exposure measured by breast milk PBDE concentrations were not observed. Roze et al. [38] reported both negative and positive neurobehavioral effects of a prenatal exposure to HBCD and several PBDE congeners among 5–6 year-old children. In a cross-sectional study on older adults [39], PBDE’s measured in the serum were not associated with performance in cognitive tasks. All associations reported in these studies were investigated using less than 150 participants.

The thyroid system is one of the targets of BFR [40]. Experimental animal studies have demonstrated that a PBDE exposure may result in a decrease of blood thyroxine [22,24,41-45] and triiodothyronine [43,44,46-48] levels. These effects were observed not only in gestation and early childhood, but also later in life [41,42]. A disruption of the thyroid system has been suggested as a mediator of the BFR neurotoxicity [9,49].

We conducted a cross-sectional study of the association between neurobehavioral function and biomarkers of exposure to BFR [serum levels of polybrominated diphenyl ether (PBDE) congeners 47, 99, 100, 153, 209, hexabromocyclododecane (HBCD), and tetrabromobisphenol A (TBBPA)] in a group of Flemish adolescents. Additionally, we investigated the association between BFR and the thyroid function as a potential biological mechanism responsible for the neurotoxicity of these chemicals.

## Methods

### Study population and data collection

The study was a part of a biomonitoring program for environmental health surveillance in Flanders, Belgium. The participants were recruited between 2008 and 2011 in two industrial areas (Genk and Menen) and from the general population of Flemish adolescents. Participants were eligible if they studied in the third year of secondary school. Hence, most participants were 14 or 15 year old, but older students were also allowed in the study.

In the general Flemish population, random sampling was attained through a multistage sampling design. In the first step, ten schools (two in each of the five Flemish provinces) - at least 20 km apart from each other - were randomly selected. In the second step, classes were randomly selected within each school and all pupils in a class were invited until the provided number of participants was reached. The number of participants per province was proportional to the number of inhabitants in that province (status at 01/01/2006).

In Genk and Menen, study areas were defined based on environmental data and the location of the industrial sites. All pupils in the requested age range living within the selected study area were eligible. Names and addresses were attained from the population registry. In Genk 54% of the adolescents were invited via a letter send to the home address and 46% during a home visit. In Menen, all participants were invited via a letter send to the home-address. Due to a small response, 30% of the invited children were contacted again via schools and 9% via a home visit.

Two weeks before the study session, subjects received two questionnaires to fill in, one for themselves and one for their parents. The questionnaire for adolescents included information about their exercising habits, amount of time spent using a computer, alcohol use, and smoking. Questions about the socioeconomic status, passive smoking and eating habits were included in the questionnaire for parents. The study session including an administration of the neurological tests, a collection of a blood sample, and a measurement of the length and the weight was around 1 hour long. Each subject received a 10 Euro voucher for the participation. Both parents and teenagers provided informed consent for participation. The study was approved by the Ethical Committee of the University of Antwerp.

The response rate equaled 22.1% in the general Flemish population, 34.3% in Genk and 22.5% in Menen. A non-responder analysis performed in a group of 106 adolescents (30 participants and 76 non-participants) did not reveal differences in socio-economic status indicators type of education (general secondary education versus other, p = 0.58), education of the father (higher education vs not, p = 0.99), or education of the mother (higher education vs not, p = 0.22) between participants and non-participants. The proportion of girls was higher among the participants (83.3% vs. 61.8% in non-participants, p = 0.03), but an equal distribution between boys and girls was a stratification criterion in the recruitment strategy. 606 adolescents participated in the study. Blood measurements were not available for three participants, and four participants did not complete any of the neurobehavioral tests. Additionally, for 84 out of the remaining 599 participants information on at least one of the covariates used in the analysis was missing. 515 subjects who completed at least one neurobehavioral test and for whom information about the covariates and serum BFR levels was available, were used in the analysis (see Table 1). This group consisted of 163 adolescents from Genk, 178 from Menen, and 174 from the general Flemish population.

**Table 1:** Descriptive statistics

**N = 515**^**A**^	
Boys	271 (52.6%)
Age, years	14.9 (0.7)
BMI, kg/m^2^	20.4 (3.1)
Type of education – general secondary	290 (56.3%)
The highest level of education of parents	
no diploma	22 (4.3%)
9 grades	52 (10.1%)
12 grades	163 (31.7%)
College of university diploma	278 (54%)
Parents owning the house	456 (88.5%)
Current smoking	65 (12.6%)
Passive smoking^B^	82 (15.9%)
Alcohol use at least once a month	129 (25%)
Number of hours a week using computer	
< 2	50 (9.7%)
2-9	271 (52.6%)
10-19	147 (28.5%)
≥ 20	47 (9.1%)
Fish consumption^C^	
Low	224 (43.5%)
Average	186 (36.1%)
High	105 (20.4%)
Physical activity in leisure time at least once a week	405 (78.6%)
Blood lipids, mg/dl	448.9 (72.9)
Blood lead, μg/dl	1.4 (0.7 to 2.9)
Sum of serum PCB 138, 153 and 180, ng/L	171.6 (58 to 445)
Thyroid hormones serum levels	
FT3, pg/mL	4.15 (0.53)
FT4, ng/dL	1.24 (0.17)
TSH, μU/mL	2.15 (0.99 to 4.45)
Neurobehavioral parameters	
Continuous Performance, reaction time, msec, N = 489	409.2 (41.8)
Continuous Performance, errors of omission, N = 489	2.3 (2.7)
Continuous Performance, errors of commission, N = 489	5.6 (3.5)
Digit Symbol, sec, N = 340	98.3 (17.7)
Digit Span Forward, N = 511	5.6 (1.03)
Digit Span Backward, N = 499	4.49 (1.01)
Finger Tapping, preferred hand, N = 511	293.7 (40.2)
Finger Tapping, non-preferred hand, N = 509	258.6 (33.8)

Arithmetic mean (standard deviation) is given for the continuous variables used on their original scale and geometric mean (5^th^ to 95^th^ percentile) for the logarithmically transformed continuous variables. Count (percent) is given for the categorical variables.

^A^Participants for whom information about the covariates, brominated flame retardants blood levels, and at least 1 neurobehavioral measure was available.

^B^At least 1 family member smoking inside the house.

^C^Based on two questions about the amount of warm and cold fish eaten per week. Low corresponds to less than 20 g, middle to 20–25 g and high to more than 25 g per day.

### Neurobehavioral tests

**Figure 1:**
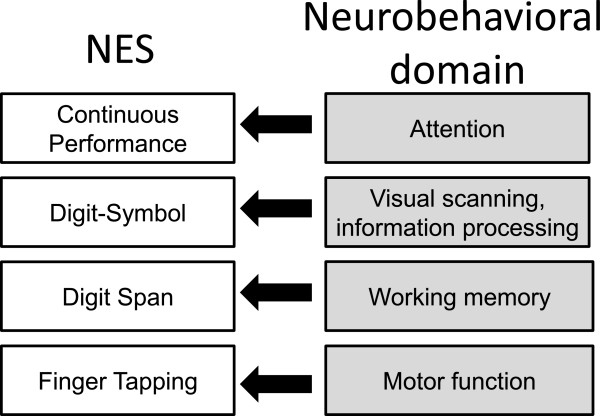
An overview of the neurological tests used in the study.

Neurobehavioral Evaluation System (NES) is a computerized battery of tests developed to study the neurological effects of an exposure to environmental agents [50]. NES has been used in a number of studies investigating the neurobehavioral impact of neurotoxicants and dose–response relationships with intensity of exposure were reported [51]. In our study, we used four tests from the NES-3 version of the test battery [52] (see Figure 1).

In the Continuous Performance test, a series of letters is displayed on the screen, one at a time, and each for approximately 200 msec. The task is to immediately respond to the letter S, and not to other letters, by pressing the spacebar. A new letter appears each 1000 msec. In total, the letter S appears 60 times. The mean reaction time for responding to the target letter in msec, the number of errors of omission, i.e., the number of times that a subject did not react within 1200 msec, the number of errors of commission, i.e., the number of responses when the letter S was not displayed, were used as the measure of performance. The test evaluates sustained attention. It showed a good test-retest reliability in a group of patients directed to a neuropsychological examination [51].

In the Digit-Symbol test, a row of 9 symbols paired with 9 digits is displayed at the top of the screen. The same 9 symbols but in a different order are shown at the bottom. When a digit is displayed, the task is to indicate the symbol, which is paired with this digit, from the bottom row. A new digit appears only after the correct symbol has been indicated. In total, 27 digits are displayed. The total time needed to complete the test measured in seconds describes the performance. The test is characterized by a satisfactory reliability [52]. In a part of the study area, a different test was administered instead of the Digit-Symbol test. As a result, the results of this test were only available for a group of 341 participants from Genk and Menen.

The Digit Span test consists of two parts. In the first part, a subject hears a sequence of digits. The task is to reproduce them. In case of a correct answer, a one digit longer sequence is presented. In case of a mistake, a sequence of the same length is presented. When two incorrect answers in a row are given, the first part of the test finishes. The second part is the same as the first one, but the sequences are reproduced in the reverse order. Digit Span Forward is the maximum span reproduced in the first part. Digit Span Backward is the maximum span reproduced in the reverse order. The first part of the test assesses the working memory span. Good performance in the second part requires both the ability to hold and manipulate information. In a part of the study area, the Digit Span test was administered using computers with touch screens. In this case the task was to indicate the digits on the screen, not using the keyboard. In order to account for a possible effect of the way the test was administered, an indicator variable was included in the regression models for this test.

In the Finger Tapping test a subject presses the spacebar as many times as possible during a trial of 10 sec. The first part of the test consists of 4 trials with the preferred-hand. The second part consists of 4 trials with the non-preferred hand. The summary measures are the total number taps with the preferred-hand and the total number of taps with the non-preferred hand. The test measures the manual motor speed.

### Blood samples analysis

PBDE congeners 28, 47, 99, 100, 153, 154, 183, and 209, HBCD, and tetrabromobisphenol A (TBBPA) were measured in the serum according to the method described by Covaci and Voorspoels [53]. Briefly, solid phase extraction was performed to prepare the samples. The eluate was purified on acid silica. The extract was further analyzed by gas chromatography mass spectrometry in electron capture negative ion mode using a 25 m × 0.22 mm × 0.25 μm HT-8 column.

Characteristics of the distributions of the BFR are shown in Table 2. PBDE congeners 28, 154 and 183 for which less than 5% of the observations had a concentration higher than the limit of quantification (LOQ), were not used in the analysis. For PBDE 47, 99, 100, 153, and 209 binary exposure indicators were used with the LOQ values as thresholds. The logarithm of the sum of PBDE 47, 99, 100, 153 was used as a measure of the total long-term PBDE exposure. In the calculation of the sum, the values below LOQ were replaced by LOQ/2.

**Table 2:** Concentrations of polybrominated flame retardants in serum (ng/L)

	**LOQ**	**Median**	**P75**	**P95**	**Max**
**BDE28**	2	<LOQ	<LOQ	<LOQ	24
**BDE47**	3	<LOQ	3	9	104
**BDE99**	3	<LOQ	<LOQ	3	12
**BDE100**	2	<LOQ	<LOQ	2	42
**BDE153**	2	2	3	8	24
**BDE154**	2	<LOQ	<LOQ	<LOQ	6
**BDE183**	2	<LOQ	<LOQ	<LOQ	5
**BDE209**	25	<LOQ	<LOQ	53	325
**HBCD**	30	<LOQ	<LOQ	59	234
**TBBPA**	15	<LOQ	<LOQ	22	186
**SUM PBDE**^A^		7	10	21	125

LOQ – limit of quantification.

^A^The sum of PBDE congeners 47, 99, 100, and 153. In the calculation, values lower than LOQ were replaced by LOQ/2.

Concentrations of PCB congeners in the serum were determined using the same method as for the BFR. The sum of PCB 138, 153, and 180 transformed logarithmically was used as an indicator of the PCB exposure. Lead was measured in the whole blood as described by Schroijen et al. [54]. Blood lipids were measured graphimetrically. Thyroid hormones FT3, FT4 and TSH were measured by competitive immune assays.

### Statistical analysis

We used SAS software version 9.2 (SAS Institute Inc, Cary, NC) for all analysis. Continuous positive-value variables with a right-skewed distribution were logarithmically transformed. Normal quantile plots of the residuals were used to examine the normality assumption for all linear models. For the individual PBDE congeners, HBCD and TBBPA the effects of the concentrations above the LOQ compared to the concentrations below the LOQ were estimated. The sum of PBDE’s was transformed logarithmically, and the effect of its two-fold increase was estimated.

In all the models investigating the effects on the neurobehavioral parameters, we corrected for age of a child, gender, type of education (general secondary education versus other), the highest level of education attained by either of the parents (using three indicator variables), whether or not the parents owned the house, smoking, passive smoking, and blood lipids. Models evaluating the effects on the number of digits reproduced in the Digit Span test were also corrected for the method of test administration (touch screen versus keyboard). Additionally, we corrected for the covariates BMI of a child, physical activity in leisure time at least once a week, computer use, alcohol use at least once a month, fish consumption, the logarithm of blood lead and the logarithm of serum PCB’s 138, 153 and 180, which were selected using a stepwise regression procedure with p = 0.15 for entering and p = 0.10 for remaining in the model. In the models investigating the effects of BFR on FT3, FT4, and TSH levels, we corrected for age, gender, BMI, and blood lipids in all models. Other variables mentioned above besides computer use were included in the model based on a stepwise regression procedure with p = 0.15 for entering and p = 0.10 for remaining in the model.

Finally, we investigated whether the effects of BFR were modified by gender by including the interaction term in the regression model.

## Results

### Characteristics of the study population

The characteristics of the study group are given in Table 1. The adolescents (52.6% boys) were between 13.6 and 17 years of age, and the mean age equaled 14.9 years. A majority of the participants (54%) had at least one parent who graduated from a college or a university. A summary of the results obtained in the neurobehavioral tests is given in Table 1.

### Determinants of neurobehavioral function

Estimates of the effects of the covariates on the neurobehavioral parameters are shown in Table 3. Gender, age, type of education, parental education, and physical activity were the most important determinants of the performance in the tests.

**Table 3:** Determinants of the neurological parameters

	**Continuous performance (N = 489)**			**Digit symbol (N = 340)**	**Digit span**		**Finger tapping**	
	**Reaction time (msec)**^**A**^	**Errors of omission**^**B**^	**Errors of commission**^**B**^	**Total latency (sec)**^**A**^	**Forward**^**A**^**(N = 511)**	**Backward**^**A**^**(N = 499)**	**Preferred hand**^**A**^**(N = 511)**	**Non-preferred hand**^**A**^**(N = 509)**
**Boys**		0.43 (0.12)**	0.29 (0.06)**	4.12 (1.82)*			9.71 (3.59)**	5.53 (3.09)
**Age, 1 year increase**	−8.57 (2.79)**	−0.14 (0.08)	−0.15 (0.04)**	−5.21 (1.21)**				5.47 (2.2)*
**BMI, IQR increase = 3.4 kg/m**^**2**^			0.06 (0.03)	2.22 (0.97)*				
**Education type – general**	−15.09 (3.76)**	−0.54 (0.11)**	−0.17 (0.06)**	−9.73 (1.82)**	0.44 (0.09)**	0.53 (0.09)**		8.12 (3.05)**
**Parents: no diploma**^**C**^								
**9 grades**^**C**^							−14.26 (5.7)*	
**12 grades**^**C**^					−0.17 (0.1)			
**Alcohol use**			0.11 (0.07)			0.19 (0.1)		
**Computer use**^**D**^	−5.26 (2.37)*							
**Physical activity**	−10.24 (4.57)*						14.72 (4.23)**	7.47 (3.61)*
**Blood lead, 2-fold increase**				3.58 (1.6)*	−0.14 (0.07)			
**Sum PCB’s, 2-fold increase**		−0.14 (0.07)*	−0.06 (0.03)		0.14 (0.05)**	0.09 (0.05)	4.44 (2.07)*	4.74 (1.79)**

*p < 0.05, **p < 0.01.

^A^ Estimated effect (standard deviation) from a linear model.

^B^ Estimated effect (standard deviation) from a negative binomial model.

^C^ Reference category: college or university diploma.

^D^The effect of a one level increase of category.

Stepwise regression estimates of the effects of covariates on the neurological parameters with p = 0.15 for entering and p = 0.10 for remaining in the model. All covariates included in one of the models are shown in the table. The set of considered covariates included: gender, age, BMI, type of education (general secondary education versus other), the highest level of education of parents (three indicator variables), whether or not the parents owned the house, smoking, passive smoking, alcohol use at least once a month, computer use, fish consumption, physical activity in leisure time at least once a week, the logarithm of blood lead and the logarithm of serum PCB’s 138, 153, and 180.

### Associations between BFR and the neurobehavioral function

**Figure 2:**
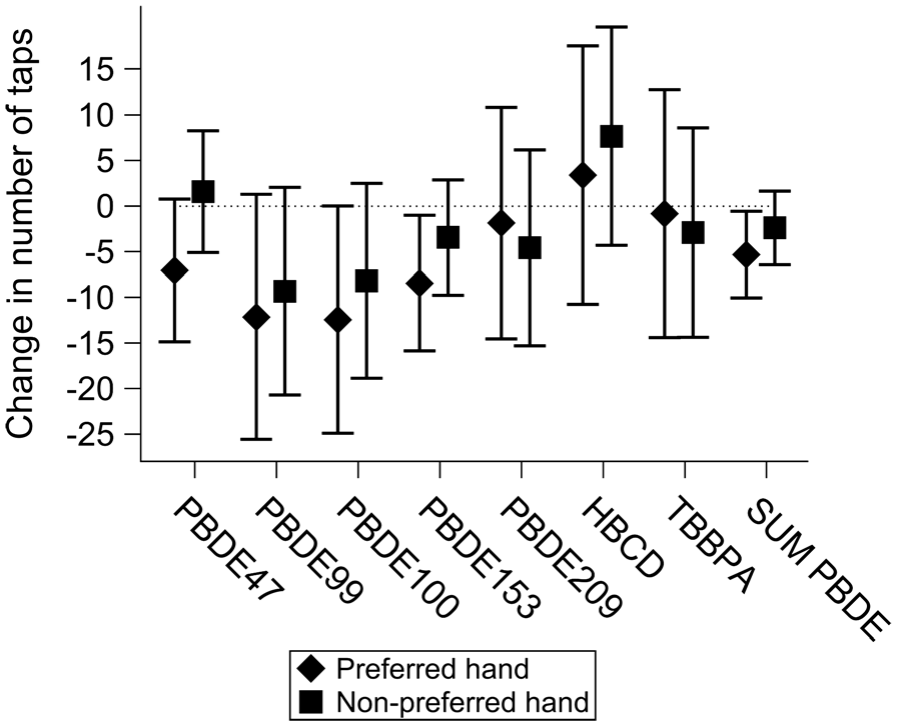
**Estimated effects of the serum levels of brominated flame retardants on the performance in the Finger Tapping test.** For the PBDE congeners, HBCD, and TBBPA the effects of levels above the LOQ were estimated. Sum of PBDE’s 47, 99, 100, and 153 was logarithmically transformed and the effects of its two-fold increase were estimated. All models were adjusted for: gender, age, type of education (general secondary education versus other), the highest level of education of parents (using three indicator variables), whether or not the parents owned the house, smoking, passive smoking, and blood lipids. Additionally, BMI, physical activity in leisure time at least once a week, computer use, alcohol use at least once a month, fish consumption, the logarithm of blood lead and the logarithm of serum PCB’s 138, 153, and 180 were included in the model based on the stepwise regression procedure with p = 0.15 for entering and p = 0.10 for remaining in the model.

We did not find any significant associations between serum levels of BFR and performance in the Continuous Performance, Digit-Symbol or Digit Span tests (Table 4). However, PBDE’s were associated with a deterioration of the performance in the Finger Tapping test in the preferred-hand condition (Figure 2). In the continuous analysis, a two-fold increase of the sum of serum PBDE’s was associated with a decrease of the number of taps with the preferred-hand by 5.31 (95% CI: 0.56 to 10.05, p = 0.029). The model explained 9.85% of the total variability and 0.87% of the variability could be attributed to the sum of serum PBDE’s. Concentrations above LOQ were associated with an average decrease of 7.04 taps (95% CI: -0.78 to 14.87; p = 0.078) for serum PBDE-47, 12.13 (95% CI: -1.3 to 25.57; p = 0.078) for serum PBDE-99, 12.43 (95% CI: -0.03 to 24.89; p = 0.051) for serum PBDE-100, and 8.43 (95% CI: 1.01 to 15.86; p = 0.026) for serum PBDE-153. The associations between serum PBDE’s and the number of taps with the non-preferred hand were usually consistent (negative association), but did not reach the level of significance. Serum HBCD and TBBPA levels were not significantly associated with performance in the Finger Tapping test.

**Table 4:** Estimated effects of serum levels of brominated flame retardants on performance in the Continues Performance, Digit-Symbol and Digit Span tests

	**Continuous Performance (N = 489)**						**Digit Symbol (N = 340)**		**Digit Span**			
	**Reaction time (msec)**^**A**^		**Errors of omission**^**B**^		**Errors of commission**^**B**^		**Total latency (sec)**^**A**^		**Forward**^**A**^**(N = 511)**		**Backward**^**A**^**(N = 499)**	
	**Effect**	**95% CI**	**Effect**	**95% CI**	**Effect**	**95% CI**	**Effect**	**95% CI**	**Effect**	**95% CI**	**Effect**	**95% CI**
**PBDE47**	3.45	−4.88 to 11.78	−10%	−29.9 to 15.6%	6.2%	−6.2 to 20.1%	−1.19	−5.57 to 3.2	−0.09	−0.29 to 0.11	−0.07	−0.27 to 0.14
**PBDE 99**	−5.39	−19.85 to 9.08	−16.3%	−46.4 to 30.8%	12.9%	−8.6 to 39.5%	−1.35	−8.94 to 6.24	0.09	−0.25 to 0.44	0.3	−0.04 to 0.64
**PBDE100**	7.61	−5.94 to 21.16	−5.8%	−37.2 to 41.4%	−3.2%	−21 to 18.5%	1.98	−5.6 to 9.56	−0.26	−0.57 to 0.06	−0.18	−0.49 to 0.14
**PBDE153**	5.09	−2.76 to 12.95	−19.3%	−36.4 to 2.3%	−2%	−12.8 to 10.2%	−1.34	−5.46 to 2.77	−0.09	−0.28 to 0.1	−0.08	−0.27 to 0.11
**PBDE209**	−1.2	−14.49 to 12.1	−17.7%	−45.1 to 23.4%	1.4%	−16.9 to 23.7%	2.08	−4.07 to 8.23	0.06	−0.26 to 0.38	−0.26	−0.57 to 0.05
**HBCD**	−3.53	−18.72 to 11.67	27.8%	−17.5 to 97.9%	21.8%	−2.5 to 52.2%	−0.44	−6.59 to 5.72	0.13	−0.22 to 0.49	−0.04	−0.39 to 0.31
**TBBPA**	−2.25	−17.28 to 12.77	−9.3%	−43 to 44.2%	−17.7%	−34.7 to 3.9%	−2.48	−10.36 to 5.41	0.03	−0.32 to 0.37	−0.05	−0.41 to 0.3
**SUM PBDE**	2.12	−2.9 to 7.13	−6.6%	−19.9 to 8.9%	0.7%	−6.6 to 8.6%	−0.39	−3.04 to 2.26	−0.01	−0.13 to 0.11	−0.04	−0.16 to 0.08

^A^ A linear model.

^B^ A negative binomial model (estimated change in%).

For the individual PBDE congeners, HBCD, and TBBPA the effects of levels above the LOQ were estimated. Sum of serum PBDE’s 47, 99, 100, and 153 was logarithmically transformed and the effects of its two-fold increase were estimated.

All models were adjusted for gender, age, type of education (general secondary education versus other), the highest level of education of parents (using three indicator variables), whether or not the parents owned the house, smoking, passive smoking, and blood lipids. Models evaluating the effects on the number of digits reproduced in the Digit Span test were in addition adjusted for the method of test administration (touch screen verses keyboard). Additionally, BMI, physical activity in leisure time at least once a week, computer use, alcohol use at least once a month, fish consumption, the logarithm of blood lead and the logarithm of serum PCB’s 138, 153, and 180 were included in the model based on the stepwise regression procedure with p = 0.15 for entering and p = 0.10 for remaining in the model.

**Figure 3:**
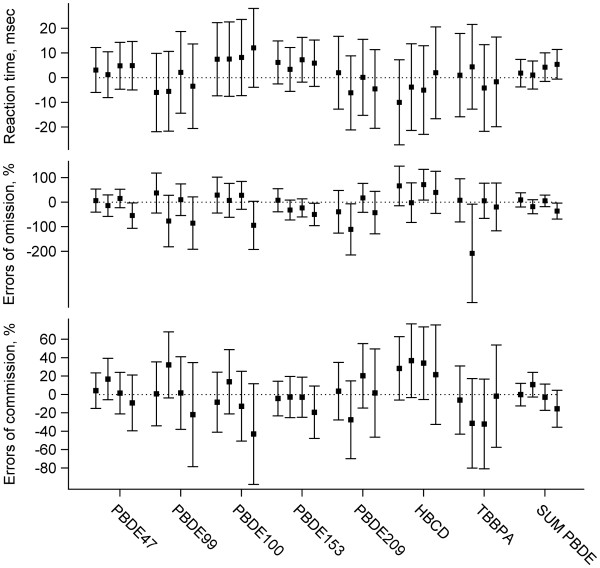
**Estimated effects of the serum levels of brominated flame retardants on the performance in the Continuous Performance from analysis with stratification by period.** For each exposure indicator, the effect on outcome in the first, the second, the third and the fourth block is shown. Each block consisted of 12 trials. The same modeling strategy as in the analysis without stratification was applied.

Also after adjusting for the number of errors of omission and commission, none of the BFR exposure indicators was significantly related with the mean reaction time in the Continuous Performance test. Exposure to BFR did not show negative associations with performance in the Continuous Performance test in analysis stratified by period (Figure 3). Gender did not significantly modify the association between the sum of PBDE’s and the number of taps with the preferred hand (p = 0.25).

### Associations with the FT3, FT4, and TSH levels

**Figure 4:**
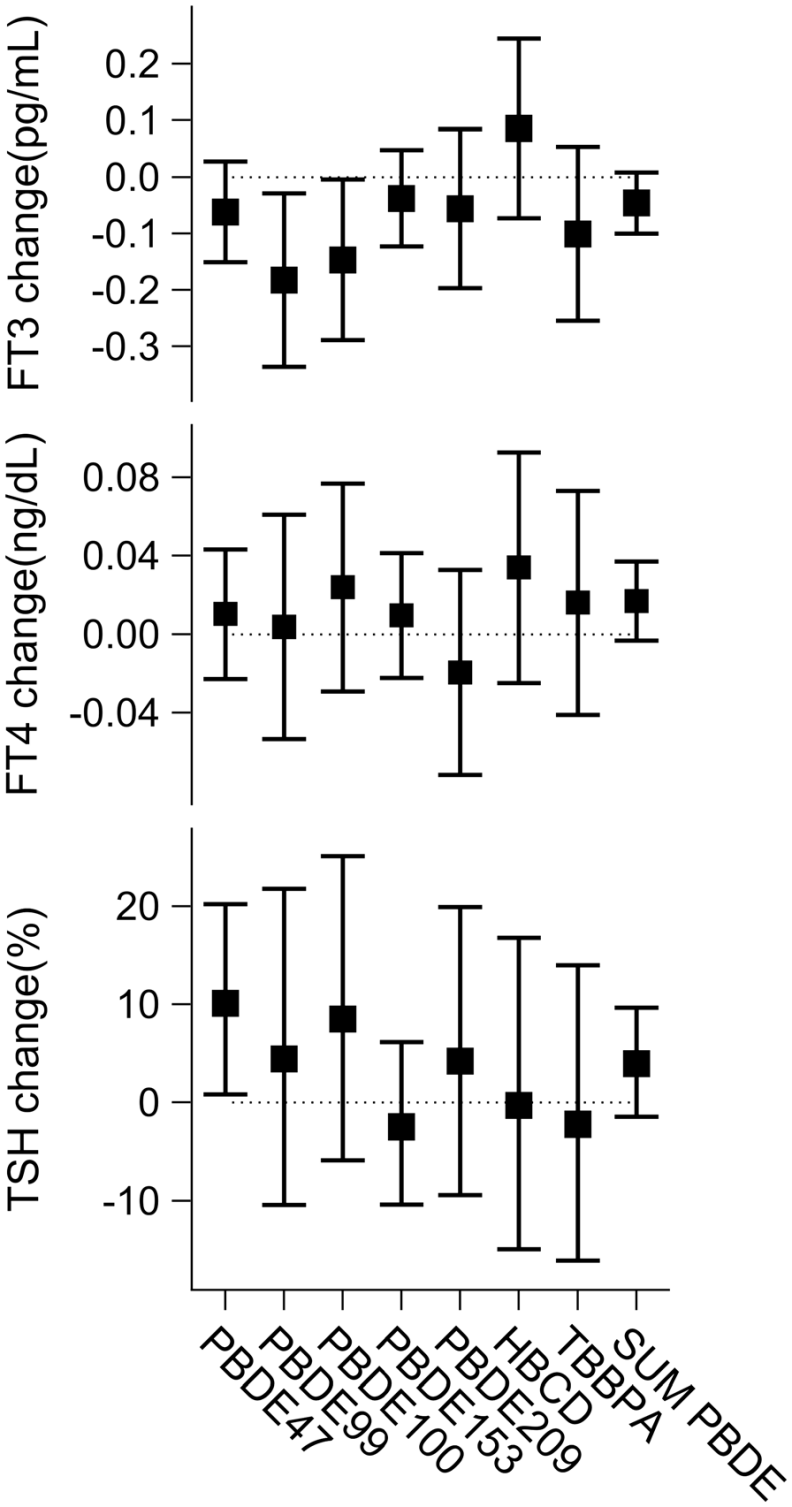
**Estimated effects of the serum levels of brominated flame retardants on serum FT3, FT4, and TSH concentrations.** For the PBDE congeners, HBCD, and TBBPA the effects of levels above the LOQ were estimated. Sum of PBDE’s 47, 99, 100, and 153 was logarithmically transformed and the effects of its two-fold increase were estimated. All models were adjusted for: gender, age, BMI and blood lipids. Additionally, type of education (general secondary education versus other), the highest level of education of the parents (using three indicator variables), whether or not the parents own the house, smoking, passive smoking, physical activity, alcohol use, fish consumption, BMI, the logarithm of blood lead and the logarithm of the sum of serum PCB’s 138, 153 and 180 were included in the model based on the stepwise regression procedure with p = 0.15 for entering and p = 0.10 for remaining in the model.

The estimated associations between BFR and FT3, FT4, and TSH serum levels after correction for possible confounders are shown in Figure 4. Serum levels above LOQ were associated with an average decrease of FT3 level by 0.18 pg/mL (95% CI: 0.03 to 0.34, p = 0.020) for PBDE 99 and by 0.15 pg/mL (95% CI: 0.004 to 0.29, p = 0.045) for PBDE-100. For the other PBDE congeners the associations had the same direction but were not statistically significant. We did not observe significant associations between PBDE congeners and FT4 levels. PBDE-47 level above LOQ was associated with an average increase of TSH levels by 10.1% (95% CI: 0.8% to 20.2%, p = 0.033). The other PBDE congeners were not significantly associated with TSH.

The continuous analyses did not show significant associations between PBDE’s and the thyroid hormone levels. For a two-fold increase of the sum of serum PBDE’s, FT3 was estimated to decrease by 0.05 pg/mL (95% CI: -0.01 to 010, p = 0.10), FT4 to increase by 0.017 ng/dL (95% CI: -0.003 to 0.032, p = 0.10), and TSH to increase by 3.9% (95% CI: -1.5% to 9.6%, p = 0.16). We did not observe any significant effects of HBCD or TBBPA on the hormone levels. The adjustment for the levels of the thyroid hormones did not substantially change the estimate of the effect of the sum of PBDE’s on the number of taps with the preferred-hand.

## Discussion

Consistently with the experimental animal studies demonstrating that exposure to PBDE’s during gestation and early childhood affects the motor function [11-22,26-28], we observed negative associations between the serum levels of these BFR and motor speed of the preferred hand in adolescents. The associations between PBDE’s and the second indicator of the motor function, the number of taps with the non-preferred hand were not significant but showed the same trend. The non-preferred hand test is performed after the preferred hand test in the Finger Tapping test. Our results resemble observations from the experimental animal studies in which a decrease of the motor activity related to PBDE exposure was present only in the beginning of the tests [11,14-21,25]. We did not observe negative associations between BFR and neurobehavioral domains other than the motor function.

Human data on the neurobehavioral effects of BFR are scarce. In the United States, a prenatal PBDE exposure was inversely associated with the level of mental development at the age of two and inteligence at the age of two and three [37]. In a Dutch study, a prenatal exposure to PBDE-47 and PBDE-99 showed a negative association with sustained attention and PBDE-153 with verbal memory measured at the age of five and six [38]. In contrast to these two studies, we conducted a cross-sectional study and focused on older children. The serum levels of PBDE’s were not associated with neurobehavioral outcomes in a cross-sectional study of older adults in New York [39]. To our knowledge, the neurobehavioral effects in adolescents have not been studied yet.

Higher PBDE-99 and PBDE-100 serum levels were significantly associated with a lower level of serum FT3, and the results for the other PBDE congeners showed the same tendency. A negative association between a PBDE exposure and the triiodothyronine concentration was also seen in some other epidemiological studies [55,56]. Consistently with the effects on FT3, most of the indicators of exposure to PBDE’s were positively related with TSH levels, although only for PBDE-47 the level of significance was reached.

Contrary to the experimental animal studies [22,24,41-45], we did not observe a negative association between PBDE’s and FT4 concentration. This is in agreement with other epidemiological studies in humans in which either non-significant associations or significant positive associations were obtained [56-59]. The discrepancy between animal and human data can be possibly explained by high PBDE doses used in the animal studies in which the effects on thyroxine levels were observed. Although we observed a positive association between PBDE-47 and TSH, the FT4 levels were not negatively associated with PBDE’s. A possible explanation is that PBDE’s may inhibit the deiodinase enzymes which serve to metabolize thyroxine to triiodothyronine [59], resulting in an increase in circulating thyroxine and a decrease in ciruculating triiodothyronine levels.

The biological mechanisms of the effects of PBDE’s on the thyroid hormones circulating in the blood have not been fully understood yet. PBDE’s exposure caused histological and morphological changes in the thyroid gland in rats indicating its decreased activity [43,60]. It also strongly upregulated uridinediphosphate-glucuronosyltransferase, an enzyme transforming molecules including thyroid hormones into excretable metabolites [44,45]. PBDE exposure also resulted in induction of pentoxy-resorufin-O-deelthylase activity [42,44,45].

Controlling for the thyroid hormone levels did not substantially change the estimated effects of the sum of PBDE’s on the motor speed. Besides the effects on the thyroid function, a disturbance of the cholinergic system may be a pathway by which PBDE’s affect the motor function. A neonatal PBDE exposure resulted in reduced or hypoactive behavioral responses to cholinergic agonist nicotine [19,61] and a decreased amount of nicotine receptors in adult rodents [17,62]. PBDE’s are also capable to disrupt calcium homeostasis in the brain, cause oxidative stress and apoptotic cell death [9,49].

Elimination of PBDE congener from human tissues depends strongly on the level of bromination [63-65]. The half-life time in human tissues has been estimated to be around 2 weeks for PBDE-209 [63-65], between a year and a few years for PBDE-47, PBDE-99, and PBDE-100 [63,65], and may be even longer for PBDE-153 [63,65]. Therefore, serum concentration of PBDE’s 47, 99, 100, 153, and the sum of these congeners’ concentration indicate to a large extent a long-term exposure. The estimated HBCD total body half-life time equaled 64 days [63]. Most of TBBPA was excreted from the body of rat within a few days after administration [66]. The serum BFR levels at adolescence which we used as exposure indicators, were unlikely to be strongly affected by the exposure during gestation and the first years of life, which may be a period of a particular susceptibility to the neurotoxicity of BFR.

The main limitation of our study was a large number of observations for which PBDE levels took values below the limit of quantification. In order to deal with this problem, we used binary exposure indicators. However, the limits of quantification which we used to create the categories did not represent critical values separating safe and dangerous exposure levels. This dichotomization of continouos exposure indicators and a possible missclassification due to the use of thresholds which did not have biological relevance might have substantially reduced the statistical power. For 222 participants the levels of both BDE-47, BDE-99, BDE-100, and BDE-153, were lower than the limit of quantification. However, the variability in the sum of these congeners observed in the rest of the participants made it an interesting measure of an overall exposure to PBDE’s. Replacing the levels below LOQ with the constant value of LOQ/2 in the calcuation of the sum introduced some measurement error. This error might have lead to an underestimation of the effect of an overall PBDE exposure.

Another disadvantage of our study is that the only aspect of the motor function we investigated was the manual motor speed, and that we assessed it only with one test. Although finger tapping is regarded a reliable measure of the motor speed [67,68], a more extended evaluation is needed to verify our findings and investigate the effects of PBDE’s on other aspects of the motor function than the manual motor speed.

Although we corrected for a number of potential confounders, we can not exclude that the associations we observed resulted from some source of confounding we failed to account for. Furthermore, a cross-sectional nature of our study does not allow to draw causal conclusions. We can not exclude the possibility that children with poor motor capabilities chose activities involving BFR exposure, which resulted in higher blood levels of these toxicants. Similarly, we can not be sure that the BFR exposure was a causing factor in the association between the toxicants and the thyroid function we observed. Our study had a fairly low response-rate. However, the comparison of socioeconomical status indicators between a subgroup of participants and non-participants did not reveal evidence of a selection bias.

## Conclusions

Our study is one of few studies and so far the largest one investigating the neurobehavioral effects of BFR in humans. Low-level PBDE exposure was associated with changes in the motor function and serum levels of FT3 and TSH. Our observations need to be further elucidated in other age groups preferably using prospectively designed studies.

## Abbreviations

PBDE: Polybrominated diphenyl ether; HBCD: Hexabromocyclododecane; TBBPA: Tetrabromobisphenol A; BFR: Brominated flame retardants; LOQ: Limit of quantification.

## Competing interests

The authors declare that they have no competing interests.

## Authors' contributions

EDH, GS, KC, LB, MK, MV, NVL, TN, and WB designed the study. EDH, GS, IS, NVL, and VN did the field work. AC and AD analyzed BFR levels. LB, MK, and TN performed statistical analysis. MK drafted the manuscript. All authors critically revised the manuscript.
